# Relationships between postural orientation and self reported function, hop performance and muscle power in subjects with anterior cruciate ligament injury

**DOI:** 10.1186/1471-2474-11-143

**Published:** 2010-07-01

**Authors:** Anna Trulsson, Ewa M Roos, Eva Ageberg, Martin Garwicz

**Affiliations:** 1 Department of Health Sciences, Division of Physiotherapy, Lund University, Sweden; 2 Department of Rehabilitation Medicine, Skåne University Hospital, Sweden; 3 Institute of Sports Science and Clinical Biomechanics, University of Southern Denmark, Odense, Denmark; 4 Department of Orthopedics, Clinical Sciences, Lund, Lund University, Sweden; 5 Neuronano Research Center, Department of Experimental Medical Science, Lund University, Sweden

## Abstract

**Background:**

Injury to the anterior cruciate ligament (ACL) is associated not only with knee instability and impaired neuromuscular control, but also with altered postural orientation manifested as observable "substitution patterns". However, tests currently used to evaluate knee function in subjects with ACL injury are not designed to assess postural orientation. Therefore, we are in the process of developing an observational test set that measures postural orientation in terms of the ability to stabilize body segments in relation to each other and to the environment. The aim of the present study was to characterise correlations between this novel test set, called the Test for Substitution Patterns (TSP) and commonly used tests of knee function.

**Methods:**

In a blinded set-up, 53 subjects (mean age 30 years, range 20-39, with 2-5 years since ACL injury) were assessed using the TSP, the Knee Injury and Osteoarthritis Outcome Score subscale sport/recreation (KOOS sport/rec), 3 hop tests and 3 muscle power tests. Correlations between the scores of the TSP and the other tests were determined.

**Results:**

Moderate correlations were found between TSP scores and KOOS sport/rec (r_s _= -0.43; p = 0.001) and between TSP scores and hop test results (r_s _= -0.40 to -0.46; p ≤ 0.003), indicating that altered postural orientation was associated with worse self-reported KOOS sport/rec function and worse hop performance. No significant correlations were found between TSP scores and muscle power results. Subjects had higher TSP scores on their injured side than on their uninjured side (median 4 and 1 points; interquartile range 2-6 and 0-1.5, respectively; p < 0.0001).

**Conclusions:**

We conclude that the Test for Substitution Patterns is of relevance to the patient and measures a specific aspect of neuromuscular control not quantified by the other tests investigated. We suggest that the TSP may be a valuable complement in the assessment of neuromuscular control in the rehabilitation of subjects with ACL injury.

## Background

Injury to the anterior cruciate ligament (ACL) is associated with knee instability, altered knee joint loading and impaired neuromuscular control, defined as the ability to produce well controlled movements through coordinated muscle activity [1-3]. All these deficiencies contribute to the development of osteoarthritis [4-6]. Moreover, alterations in dynamic multi-joint stabilization, an ability fundamental to any weight-bearing movement, have been described in subjects with ACL injury [1,7-9].

Function in subjects with ACL injury may be evaluated with self-reported outcome scores [10,11], various forms of hop tests [3,12-15] and with dynamometry [16-19]. The World Health Organization (WHO) advocates that instruments developed and used for the assessment of body functions and activities should be of relevance to the patient and put in the perspective of subjects' perceived participation (International Classification of Functioning, Disability and Health, ICF) [20]. In accordance with the ICF, the above mentioned tests pertain to the domains of body function and structure, activity and participation, hence describing not only important aspects of a subject's knee function, but also the consequences for the individual. However, although these commonly used tests involve all ICF domains they do not reflect alterations in dynamic multi-joint stabilization, despite the fact that this is of special importance in joints with both mechanical and dynamic instability, as in subjects with ACL injury [2,9]. Moreover, both hop tests and muscle power tests mainly measure parameters such as distance, height and power, although it is known that persistent changes in neuromuscular control cannot be detected by such measures alone [21].

To enable the assessment of dynamic multi-joint stabilization, we recently embarked upon the development of a new observational test set measuring dynamic joint stability as the ability to stabilize body segments in relation to each other and to the environment (postural orientation [22]) during weight-bearing movements [23]. An alteration in this ability is defined as a substitution pattern. The first evaluation of the test set, called the Test for Substitution Patterns (TSP), showed that subjects with ACL injury display more frequent and/or more clearly present substitution patterns on their injured, and to a lesser extent also on their uninjured side, than do uninjured controls. Moreover, substitution patterns could be detected not only in the region of the injured knee joint but also in the region of adjacent joints [23].

The main aim of the present study was to characterise the relationships between TSP scores and a patient-relevant outcome score (Knee Injury and Osteoarthritis Outcome Score, KOOS, subscale sport/recreation (sport/rec) [10]), between TSP scores and 3 hop tests [12] and between TSP scores and 3 muscle power tests [16]. We also compared TSP scores for subjects' injured and uninjured sides. Since the TSP is designed to measure postural orientation, a complex aspect of neuromuscular control that should be of relevance to subjects' self-reported function, we hypothesized that TSP scores would show some correlation to KOOS sport/rec scores. For the same reason, we also expected to find some correlation between TSP scores and hop test results, since hop performance requires a certain level of complex coordinated muscular activity. In contrast, we did not expect to find any correlation between TSP scores and muscle power results, since muscle power *per se *represents the ICF domain body function and structure, not requiring the same amount of complex coordinated muscular activity. Nevertheless a comparison between TSP and muscle power test results was of interest since muscle power testing is very often used in the evaluation of individuals with ACL-injury.

## Methods

This double-blind, cross-sectional study was approved by the Research Ethics Committee of Lund University. All subjects gave their written informed consent to participate.

### Subjects

The 53 subjects with ACL injury included in the present study were from a cohort of 54 subjects with ACL injury, with and without surgical reconstruction, included in a cross-sectional study on muscle power and functional performance [18]. These 54 subjects were, in turn, part of a subgroup of 121 subjects with ACL injury included in a randomized controlled trial (RCT) comparing the outcome of training and surgical reconstruction versus training only [24].

The inclusion criteria for the RCT were: complete ACL rupture, age 18-35 years, a moderate to high level of physical activity (corresponding to 5-9 on the Tegner Activity Scale, which ranges from 0 (least strenuous activity for the knee) to 10 (most strenuous activity for the knee) [25]). All subjects followed a moderately aggressive training programme for at least 4 months, supervised by physiotherapists. The original 121 subjects in the RCT were investigated within four weeks of injury (for details see [24]).

Of the 121 subjects in the RCT, 92 fulfilled the inclusion criteria of 2-5 years since injury at the time of the present investigation. Four subjects were excluded; one due to pregnancy, and 3 subjects because they had used crutches during the past 3 months. Two subjects could not be reached, 18 subjects declined to participate, 8 cancelled the appointment due to reasons unrelated to their injury, and 6 subjects did not show up for the assessment, leaving 54 patients for assessment (for details see [18]). One of these 54 subjects declined to perform the TSP test, yielding 53 subjects, of which 15 were women (Table 1).

**Table 1:** Characteristics of the 53 subjects

Characteristic	
Age, mean ± SD (min-max) years	30 ± 5.2 (20-39)
Women, n (%)	15 (28)
BMI, mean ± SD kg/m^2^	24.6 ± 3.4
Surgically reconstructed, n (%)	36 (68)
Injured right knee, n (%)	27 (51)
>2-year old contralateral ACL injury, n (%)	11 (21)
Tegner activity level, median (quartiles)	4 (2, 6)

Data in the present study were collected at a mean of 3 years (SD 0.9, range 2-5 years) after injury, and at the same time as the study on muscle power and functional performance [18], and are not part of the RCT protocol. The outcome of the RCT will be presented separately.

### Procedure

Subjects were assessed using the TSP, KOOS sport/rec, 3 hop tests [12] and 3 muscle power tests [16]. Subjects were encouraged to continue their daily activities as usual before their test session, but not to participate in any strenuous activity the day before. The tests were performed in the following order, with the right leg being tested first and with an interval of only a few minutes between the tests: 1) the TSP; 2) 5 minutes of warm-up (stationary cycling, squats, toe rises and jumps); 3) vertical jump, one-leg hop test for distance and side hop; 4) knee extension power test, knee flexion power test and leg press power test (these in a randomized order). Subjects were dressed in shorts and T-shirt. While performing the hop tests and muscle power tests and the subtest "Forward lunge from stairs" in the TSP, they also wore trainers.

All tests were performed in a blinded manner. The examiner was given no information on which leg was injured, and had no knowledge of the subjects' Tegner scores. Tubi-grip^® ^stockings (MEDLOCK Medical, Oldham, UK) covered both knee joints to hide possible scars from knee surgery. The subjects were given no information on what the examiner was observing or scoring during the five subtests (see below) included in the TSP.

### The TSP

The TSP has been described in detail previously [23]. In brief, it is an observational test set evaluating the ability to maintain an appropriate relationship between the body segments, and between the body and the environment when performing weight-bearing movements [22]. The focus is on the detection of predefined substitution patterns in the legs, trunk, arms and/or neck, such as: 1) increased pronation of the supporting foot compared to standing on two legs; 2) knee medial to the supporting foot (knee not in line with hip and foot); 3) lateral displacement of the hip-pelvis region on the supporting side; 4) displacement of the trunk (for instance, forward bending or lateral displacement of the trunk) on the supporting side; 5) displacement of the arms; 6) shorter stride; 7) increased support from the hands, or taking a more careful stride on one of the two sides; 8) avoidance of weight bearing on the back leg during return in forward lunge and/or 9) displacement of body weight to either side. Note that, the same substitution pattern can be observed in more than one subtest - see just below.

The first evaluation of the TSP consisted of 9 subtests [23]. Five of these subtests showed an ability to discriminate between patients and uninjured controls in that study, and were therefore used in the present study: "Body weight-altering test" (including substitution patterns 1, 2, 3, 4 and 5), "Tip-toe standing knee flexion" (including substitution patterns 2, 3, and 4), "Knee flexion-extension standing on one leg" (including substitution patterns 1, 2, 3, and 4), "Forward lunge from stairs" (including substitution patterns 2, 4, 6, 7 and 8), and finally "Mini-squat" (including substitution pattern no. 9), yielding a total of 18 possible substitution patterns. The subtests were demonstrated by the same examiner, who also gave standardized instructions to the participants. The subtests were performed in a random order; each subtest being performed five times in succession.

The presence of substitution patterns during the 5 subtests was scored for each leg separately using a four-point, ordinal scale (0-3), where "0" denotes no substitution pattern present; "1" denotes substitution pattern possibly present; "2" denotes substitution pattern clearly present; and "3" denotes subject performed very poorly (for example not able to perform the predefined number of times or with no similarity to the task). The scores 1-3 were awarded when the substitution pattern was observed in at least three out of the five times the subtest was performed.

Note that the TSP total score for an individual subject is the sum of the scores awarded for all five subtests, and has a possible range from 0 points (indicating no substitution pattern present) to 54 points (3 points × 18 substitution patterns).

### KOOS

The KOOS [10], Swedish version [26], was used for subjects' ratings of their knee symptoms. The KOOS is a disease-specific, self-administrated questionnaire with 42 questions in 5 subscales (pain, symptoms, activities of daily living, sport and recreation, and quality of life), with a scale extending from 0, indicating extreme problems, to 100, indicating no problems. In this study, the KOOS subscale sport/rec was used because of its relevance to hop performance, muscle power and postural orientation (Table 2).

**Table 2:** Scores for KOOS sport/rec, hop tests and the muscle power tests for the subjects' injured side

KOOS sport/rec	Hop test			Muscle power		
	Vertical jump (cm)	One-leg hop (cm)	Side hop (n)	Knee extension (W)	Knee flexion (W)	Leg press (W)

75 ± 3.5	16.1 ± 0.6	131.1 ± 3.3	36.3 ± 2.0	239.1 ± 11.3	164.3 ± 7.5	476.1 ± 20.0

Values given are means ± SE, n = 53. KOOS sport/rec = Knee Injury and Osteoarthritis Outcome Score, subscale sport and recreation.

### Hop tests

The hop tests used in the study were the vertical jump, the one-leg hop and the side hop, and were performed according to Gustavsson et al. [12], described in detail previously [18]. In brief, subjects attempted to maximize the jump height while performing 3 approved trials for the vertical jump. The height of the jump was measured by a computerized system (Muscle Lab; Ergotest Technology, Oslo, Norway) using a field of infrared light (~10 mm above the floor) to measure the flight time. The height of the jump (cm) was then calculated by the system. The one-leg hop for distance was performed taking off and landing on the same foot, with the hands placed on the back, and was measured by the test leader from the big toe at the push-off to the heel at landing (cm). Side hops were performed as the maximum number of side hops on one leg during a period of 30 seconds. The subjects jumped from side to side outside 2 parallel tape-strips 40 cm apart. The jumps were videotaped and the number of successful hops on each leg was recorded. The best results for each leg in each test were used in the analysis.

### Muscle power tests

The muscle power tests were performed according to Neeter et al. [16] and have previously been described in detail [18]. Briefly, the tests were performed in weight training machines, where the average power was calculated by a computerized muscle function measuring system (Muscle Lab, Ergotest Technology) with 5 maximum trials at 5 weight levels. The knee extension power test was performed from ~110° of knee flexion to full knee extension, on one leg at a time, and was chosen to reflect quadriceps muscle power (open chain exercise). The knee flexion power test was performed from full knee extension to ~110° of knee flexion, chosen to reflect hamstring muscle power (open chain exercise), on one leg at a time. The leg press power test was chosen to reflect lower extremity muscle power during leg press (closed chain exercise), and was performed with a starting position of ~90° of knee and hip flexion and a final position with the knee in full extension, on one leg at a time.

### Statistics

The TSP is scored on an ordinal scale and the median and range were therefore used to characterise the data. All calculations and statistical analyses were carried out using SPSS version 15.0. In the within-group comparisons, the Wilcoxon signed rank test was used, and in the between-group comparisons, the Mann-Whitney U test was used. P-values less than or equal to 0.05 were considered statistically significant. Spearman's rank correlation coefficient was used as a measure of correlation.

There were no statistically significant differences in the TSP total scores for the surgically reconstructed and non-surgically treated subjects. This was true for both the subjects' injured side: median total score 3.5 points (range 0-17) for reconstructed subjects, and 4.0 points (range 0-18) for non-surgically treated subjects (p = 0.88), and for the uninjured side: median total score 1 point (range 0-8) and 1 point (range 0-4), respectively (p = 0.76). Since this was the case, the data from all subjects were pooled together in the analysis, irrespective of whether they had undergone ACL-reconstruction or not.

## Results

### TSP scores

**Figure 1:**
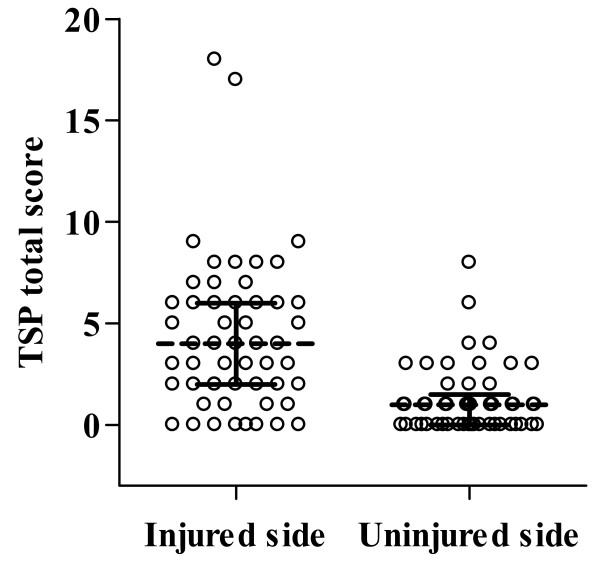
**Scatter plots for the TSP total score for subjects' injured and uninjured sides**. The horizontal lines indicate the median and interquartile range, n = 53.

On the subjects injured side, irrespective of whether the knee had undergone ACL-reconstruction or not, there was a higher TSP total score, median 4 points (interquartile range 2-6), than on the uninjured side, median 1 point (interquartile range 0-1.5) (p < 0.0001) (Figure 1). The TSP total score in this study ranged from 0-18 points for the injured side and from 0-8 points for uninjured side.

**Figure 2:**
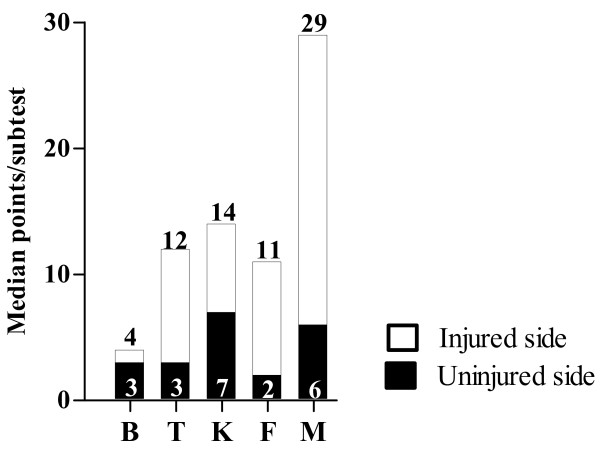
**Median score (points) for each subtest in the TSP for injured and uninjured sides**. B = Body-weight-altering test, T = Tiptoe-standing knee flexion, K = Knee flexion - extension, F = Forward lunge from stairs, and M = Mini-squat, n = 53.

Statistically significant differences were also found between the injured and uninjured sides for the five different subtests in the TSP (p < 0.001 to p = 0.008). Figure 2 shows the median values for each of the five subtests, for the injured and uninjured sides. Eight subjects (15%) showed no substitution patterns on their injured side, while 22 subjects (42%) showed no substitution patterns on their uninjured side.

### KOOS sport/rec, hop test and muscle power test results

Mean values of the scores obtained with the KOOS sport/rec, hop test and muscle power test are presented in Table 2.

### Relationships between TSP, KOOS sport/rec, hop test and muscle power results

**Figure 3:**
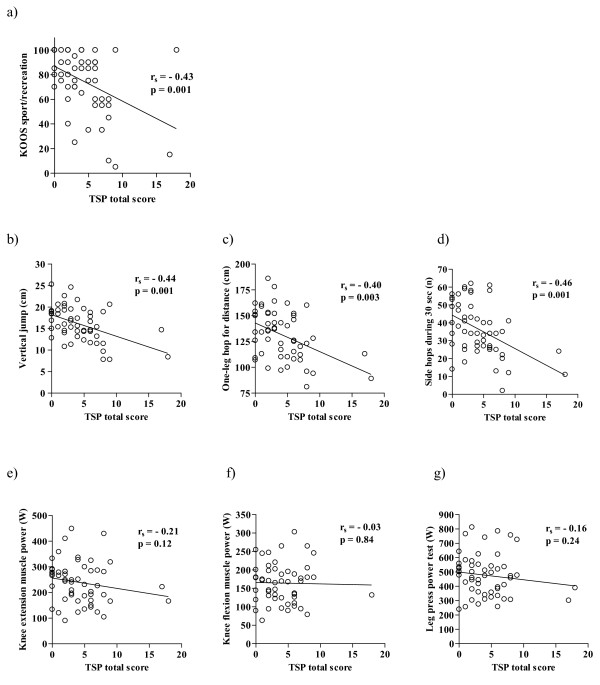
**Correlations between TSP total score and KOOS sport/rec (a), hop tests (b-d) and muscle power tests (e-g), for the injured side**. KOOS sport/rec = Knee Injury and Osteoarthritis Outcome Score, subscale sport and recreation, r_s _= Spearman's rank correlation coefficient. TSP = Test for Substitution Patterns. n = 53.

The scatter plots in Figure 3a-g show the relationships between the scores obtained with the TSP, KOOS sport/rec, hop tests and muscle power results for each subject. Spearman's rank correlation coefficients (r_s_) were calculated for all comparisons and are given in the individual diagrams. Moderate correlations were observed between TSP scores and KOOS sport/rec scores (r_s _= -0.43, Figure 3a) and between TSP scores and hop test results (r_s _= -0.40 to -0.46, Figure 3b-d), indicating a higher extent of substitution patterns being correlated to worse self-reported sport and recreation function and worse hop performance. In contrast, no significant correlations were seen between TSP scores and muscle power results (Figure 3e-g).

## Discussion

The main findings of the present study were moderate correlations between TSP scores and KOOS sport/rec and between TSP scores and hop test performance, but no significant correlations between TSP scores and muscle power results. These findings indicate that the TSP is of patient relevance, and reflects a specific aspect of neuromuscular control not quantified by the other tests investigated. The patients had a higher TSP total score on their injured side than on their uninjured side, in accordance with previous findings.

As hypothesized, the results of this study indicate that the TSP measures aspects of neuromuscular control not quantified by commonly used self-reported questionnaires, hop tests and muscle power tests. We suggest that the TSP reflects and quantifies the *quality *of a movement with respect to dynamic joint stability [1] and postural orientation [22]. The fact that this qualitative aspect of performance is not evaluated in hop tests could be one reason why only a moderate correlation was found between TSP scores and the hop test results. Although muscle power is a prerequisite for movement, other abilities such as dynamic joint stabilization, coordination and balance, are also necessary to perform complex movements such as those in the TSP in a well controlled way. This could be one of the reasons of the lack of correlation between the TSP scores and the muscle power results. In particular, the association between lower extremity muscular strength and hop test results in individuals with ACL injury or ACL reconstruction has previously been found to be low to moderate [27].

According to the ICF, muscle power tests pertain to the domain of body function and structure, while hop tests and the TSP pertain to the domain of activity. Although it could be expected that tests within the same ICF domain should be highly correlated, the moderate correlations found between the TSP and hop tests demonstrate that they only partly measure the same neuromuscular ability, and therefore cannot be considered interchangeable. The KOOS subscale sport/rec, which was chosen because of its relevance to hop performance, muscle power and postural orientation, showed a moderate correlation to the TSP. This implies that the TSP, that we consider to be of patient relevance because of a low occurrence of substitution patterns associated with less self-reported difficulty in the KOOS sport/rec scale, nevertheless captures an aspect of neuromuscular control not assessed by the KOOS sport/rec.

The Lysholm knee scoring scale [25] was not used in the present study. The main reason for this is that the sensitivity of the Lysholm scale in detecting functional limitations in patients with ACL injury, compared with other diagnostic groups, has been questioned (and may therefore have limited validity) [28], but also because the Lysholm scale has been reported to not accurately identify problems during strenuous activities [15]. Furthermore, Hoher and co-workers found that self administration of the Lysholm score yielded worse scores than completion by an observer [29]. KOOS (sport/rec), on the other hand, is a more relevant instrument regarding the aims and issues in the present study because it is a patient-centred instrument, while the Lysholm knee scoring scale focuses on the perspective of the operating surgeons and orthopaedic measurements.

The subjects with ACL injury in this study did not constitute a homogeneous group. Almost 70% (Table 1) had been treated with reconstruction and training, while the rest underwent training only, some may have had mechanical instability, others not, and some might have been copers while others were non-copers. However, since the main focus of this study was the relation between the TSP and self-reported function, hop performance and muscle power, and not between different groups of patients, these aspects will have had little or no effect on the results.

In this ACL-injured cohort there seemed to be no statistically significant difference in TSP total scores between surgically reconstructed and non-surgically treated subjects (see Methods). Although not conclusive, this finding is in line with the previous results of Ageberg et al., who reported that reconstructive surgery was not a prerequisite for restoring muscle function [18]. It is not yet known what causes changes in postural orientation, manifested as substitution patterns in ACL-injured subjects. One may speculate that an important contributing factor is a change in the proprioceptive input from the joints, muscles and ligaments [2,9,30,31], leading to altered information processing in the spinal and supraspinal sensorimotor circuits [9,30,32]. This change in information processing could result in inadequate efferent motor output, in turn causing defective joint stability which, together with mechanical instability, is manifested as an alteration in the position of the knee in relation to the hip and foot [33], i.e. the observed substitution patterns. Inappropriate control of the muscles acting on adjacent joints has also been observed, for example, as a disturbance in the activation of the gluteus maximus in subjects with recurrent ankle ligament injuries [34,35]. Taken together, these facts underline the complexity of neuromuscular control, and despite the fact that the sensorimotor aspect of the maintenance of postural orientation is of great importance, it is not reflected by the test instruments commonly used in the rehabilitation of ACL-injured subjects. Further investigations of the underlying mechanisms of substitution patterns are therefore essential, and are already in progress.

Since a difference is often seen between injured and uninjured sides in hop tests and muscle power tests, the higher TSP total scores for the subjects' injured side found in the present study are in line with previous findings [18]. Yet, the TSP is not intended as a diagnostic test for ACL injury. Instead, its main purpose is to provide information on subjects' postural orientation in different weight-bearing positions resembling both conditions in daily life and more strenuous activities where the dynamic stability of the joint is challenged. The TSP could therefore be of use primarily for the physiotherapist in the identification of impaired neuromuscular control after ACL injury, when planning and carrying out training and rehabilitation without unfavourable substitution patterns, but also when deciding the appropriate time to return to activity and sports after an ACL injury.

Several factors such as validity, generalizability and reliability [23] must be further investigated before the TSP can be used in the clinic. Some limitations of the present study in this regard should be pointed out. The time after injury, 2-5 years, was chosen since ACL-injured individuals have been reported to have the best possible muscle function and self-reported outcomes at this point in time [3,4]. However, the relationships between TSP scores, hop tests and muscle power tests may change over time. It is not clear, for instance, how the presence and/or severity of substitution patterns would be affected by decreased muscle power and hop ability. A longitudinal study must be performed to address this question. Nevertheless, the fact that the subjects in the cohort studied here still displayed substitution patterns, despite having undergone a moderately aggressive training programme under the supervision of physiotherapists for at least 4 months, may suggest that substitution patterns are not easily corrected, even with focussed training, and that they do not disappear over a period of 2-5 years.

In the first study on the TSP, 9 subtests were assessed [23]. Five of these subtests were used in the present study, based on their potential to discriminate between patients and uninjured controls in the former study. These subtests showed statistically significant differences between the injured and uninjured sides in the ACL-injured cohort. It could thus be argued that these five subtests are sufficient to discriminate between injured and uninjured sides or subjects, but further studies are required to finally settle this matter. Nevertheless, the TSP has now been used in two different cohorts with ACL-injured subjects, and significant differences have been found in TSP total scores and in each subtest between subjects' injured and uninjured sides, as well as between ACL-injured subjects and controls. The two ACL-injured cohorts included subjects who had and who had not undergone surgical ACL reconstruction, as well as subjects in a well rehabilitated stage. Also, both men and women were included in the two cohorts. The TSP has not, on the other hand, been evaluated in recently ACL-injured subjects. One could speculate that a higher TSP total score may be found in the recently injured due to greater neuromuscular impairment and, therefore, the discrimination between injured and uninjured sides or subjects would be even better. Furthermore, it may be possible to apply the TSP to subjects with other injuries to the knee than ACL injury, such as meniscus injury and/or cartilage damage, since Roos et al. found no difference in self-reported difficulty in KOOS sport/rec between subjects with ACL injury, meniscus injury, cartilage damage or a combination of these injuries [26].

Last but not least, to further understand the significance and underlying mechanisms of substitution patterns, and to devise appropriate interventions for reducing these patterns, further studies of, for example, reflex contraction latency of muscles measured with electromyography, documentation with a computerized motion capture system, and longitudinal studies of how substitution patterns change over time, would be highly valuable.

## Conclusions

We conclude that the Test for Substitution Patterns is of relevance to the patient, and that it measures a specific aspect of neuromuscular control not quantified by the other tests investigated. We therefore suggest that the TSP may be a valuable complement in the assessment of neuromuscular control in the rehabilitation of subjects with ACL injury.

## Competing interests

The authors declare that they have no competing interests.

## Authors' contributions

AT contributed to the conception and design of the study and the acquisition of data; she performed the data analysis, and was in charge of drafting and writing the manuscript. ER contributed to the conception and design of the study and the acquisition of data and gave intellectual feedback on the manuscript. EA contributed to the conception and design of the study, the acquisition and analysis of the data, and gave intellectual feedback on the manuscript. MG contributed to the conception and the design of the study, the analysis of the data, contributed in drafting of the manuscript and gave intellectual feedback on the manuscript. All authors read and approved the final manuscript.

## Pre-publication history

The pre-publication history for this paper can be accessed here:

http://www.biomedcentral.com/1471-2474/11/143/prepub
